# A Reliable and Real-Time Tracking Method with Color Distribution

**DOI:** 10.3390/s17102303

**Published:** 2017-10-10

**Authors:** Zishu Zhao, Yuqi Han, Tingfa Xu, Xiangmin Li, Haiping Song, Jiqiang Luo

**Affiliations:** 1 School of Optoelectronics, Image Engineering & Video Technology Lab, Beijing Institute of Technology, Beijing 100081, China; nicholasldm@126.com (Z.Z.); li_xiangmin@bit.edu.cn (X.L.); luojiqiang@yeah.net (J.L.); 2 Beijing Key Laboratory of Embedded Real-Time Information Processing Technique, School of Information and Electronics, Beijing Institute of Technology, Beijing 100081, China; yuqi_han@bit.edu.cn; 3 Key Laboratory of Photoelectronic Imaging Technology and System, Ministry of Education of China, Beijing 100081, China; 4 China North Vehicle Research Institute, Beijing 100081 China; hpsong@noveri.com.cn

**Keywords:** visual tracking, salient prior, color distribution, high-confidence update strategy, research mechanism, scale pyramid

## Abstract

Occlusion is a challenging problem in visual tracking. Therefore, in recent years, many trackers have been explored to solve this problem, but most of them cannot track the target in real time because of the heavy computational cost. A spatio-temporal context (STC) tracker was proposed to accelerate the task by calculating context information in the Fourier domain, alleviating the performance in handling occlusion. In this paper, we take advantage of the high efficiency of the STC tracker and employ salient prior model information based on color distribution to improve the robustness. Furthermore, we exploit a scale pyramid for accurate scale estimation. In particular, a new high-confidence update strategy and a re-searching mechanism are used to avoid the model corruption and handle occlusion. Extensive experimental results demonstrate our algorithm outperforms several state-of-the-art algorithms on the OTB2015 dataset.

## 1. Introduction

Visual tracking is one of the fundamental tasks in computer vision, with a plethora of applications such as video surveillance, robotics, human-computer interaction, etc. Despite the significant progress in recent years, designing a generic tracker is still rather challenging because of several challenges, including illumination variations, scale variations, fast motion, partial or full occlusion, background clusters and so on. Most trackers suffer from performance degradation due to those problems, and often gradually drift from the target.

In this paper, we attempt to develop a reliable and real-time tracking method based on a milestone tracker known as STC [1]. STC is a simple and effective correlation-based tracking method proposed in 2014, which aims at learning a filter to locate the target by identifying the maximum response of the samples and training templates. Such correlation filter (CF)-based trackers can tacitly take advantage of FFT operations. Benefitting from this, the STC tracker achieves rates of 350 frames per second with good performance.

Besides that, the STC tracker utilizes the spatio-temporal relationships between the background and the target in sequential frames. In earlier tracking approaches, context information has been utilized in visual tracking. These trackers extract the information around the target, using descriptors like SURF to describe these regions however, these trackers ignore the temporal information and most of them are complicated in calculation due to the training procedure. In contrast to this development, the STC tracker learns a prior possibility model instead of training samples, in order to reduce the computational cost. To be more specific, the STC tracker models the statistical relationship between the simple low-level features from the target and its surrounding regions. The tracking problem is then posed by computing a confidence map, which takes consideration of the prior information of the target location and effectively alleviates the location ambiguity. Due to the favorable simplicity of its concise framework, STC tracker is well suited for time-critical tracking applications. Hence, in this paper, we follow its original framework while enhancing its performance in several aspects as described in the following paragraphs.

**Figure 1:**
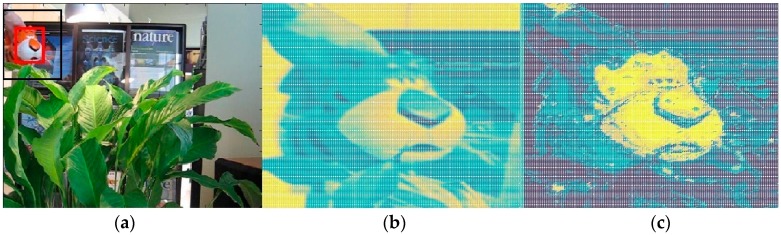
The salient prior model we used with color distribution. (**a**) The original frame. (**b**) The STC prior model obtained by grayscale feature. (**c**) The proposed salient prior model by color distribution. One can see that the difference between the target and its surrounding regions is much more obvious in the RGB channel than the one of grayscale feature.

Wang et al. [2] break a modern tracker down into five constituent parts, namely, the motion model, feature extractor, observation model, model update and ensemble post-processor to better understand and diagnose a visual tracking system. According to their research, robust feature representation plays a significant role in visual tracking. However, in the traditional STC tracker, the authors employ grayscale features to build the context prior model. We argue that compared with color features, the gray-scale feature may lose considerable visual information, which would reduce the discrimination of the prior model and even lead to failure. On the other hand, considering the time-sensitive nature of tracking applications, running speed is a crucial indicator to evaluate a tracker. Hence, in this paper, we advocate a salient prior model based upon a multi-channel color histogram, as shown in Figure 1, to represent the target and the surrounding background more robustly with a low computational burden.

Our second contribution is ameliorating the scale adaptive mechanism. The STC tracker assumes that the scale of the target could be represented by the max response in a response map. However, the response value is not stable and may change abruptly due to the complex background and tracking conditions, such as illumination variations, background clutter and so on. Based on the analysis above, it is unreasonable to use the peak value of the response map to estimate scale variation.

In our algorithm, we employ a scale pyramid to estimate the target’s scale which is independent of the response map after obtaining the target location. Experiments show that this mechanism is simple but effective. Besides that, like other CF-based trackers, the STC tracker uses a linear interpolation to update the model each frame. However, such a radical mechanism would collect previous mistakes and noise examples when the target undergoes occlusion, and would hardly recover from the tracking drift due to the contamination of the model. To handle this problem, we apply novel response map indicators to guide the tracker for high confidence updates and to predict the drift. Finally, a re-search strategy is proposed to handle the problem of the target drift and occlusion. We evaluate our algorithm on the OTB dataset and the experiments show that our algorithm achieves good performance.

The rest of our article is organized as follows: in Section 2, we review research work related to ours. In Section 3, we introduce the proposed approach, namely the salient prior model, scale adaption scheme, high confidence updating model and re-detection strategy in details. Numerous experimental results and analysis are shown in comparison with other state-of-the-art tracking algorithm in Section 4.

## 2. Related Works

In this section, we briefly review the development of tracking methods and list works closely related to our algorithm. The essence of object tracking is to estimate the location of an initialized target in each frame of a video sequence. Most existing trackers adopt either a generative or a discriminative approach. In generative trackers, an elaborate appearance model is often designed to describe a set of target observations in order to search the best-matching patches for the tracked object [3,4]. To solve the problem of appearance changes of the target, the incremental learning for robust visual tracking (IVT) learns an incremental subspace model [5]. Kwon et al. [6] used an observation model composed of multiple basic models to adapt to appearance changes. In recent years, sparse representation-based trackers have achieved great progress. This kind of algorithm uses a sparse dictionary to improve the efficiency and make the trackers more robust. Liu et al. [7] improved the mean-shift algorithm with a local sparse model. Xue et al. [8] cast the tracking problem as finding a sparse approximation in a template subspace.

On the other hand, discriminative trackers employ machine-learning techniques to train an appearance model based on samples of the target and its background. With the use of background information, in recent years, discriminative algorithms have often yielded better performance than generative ones. Multiple instance learning (MIL) [9,10] was proposed by Babenko et al. which adopts bag labels to select features. Kalal et al. [11] proposed a P-N learning tracker which classifies samples as positive and negative for learning tracking classifiers. The tracking-learning-detection method (TLD) [11] uses a sampling strategy with structural constraints for real-time visual tracking. Zhang et al. [12] used the compressive features to train a Naïve Bayes classifier. Struck [13] is a tracker which links the target’s location space with the training samples by a structured Support Vector Machine (SVM), however, the number of training candidates limits the efficiency of such an approach. To overcome the above issues, Bolme et al. introduced the correlation filter into tracking [14]. Correlation filter-based trackers utilize a learned filter to localize the target in each frame by identifying the maximal correlation response of the template and input patches in a search window. Henriques et al. [15] used circular image patches to train the correlation filter in kernel space with low computation burden. Several follow-ups have been proposed to enhance the robustness and the efficiency of CF- based trackers [16,17,18]. In the CN tracker [18], color attributes are added to the CSK framework [19] , and an adaptive dimension reduction technique is proposed, which demonstrates the importance of color features in visual tracking. In the paper proposed by Martin et al. [16] and the paper proposed by Li et al. [17], the authors handle the scale variation by designing a three-dimensional filter and scale pyramid with several different scales, respectively. In STC [1], the authors presented a simple yet fast algorithm which exploits the dense spatio-temporal context for visual tracking.

**Figure 2:**
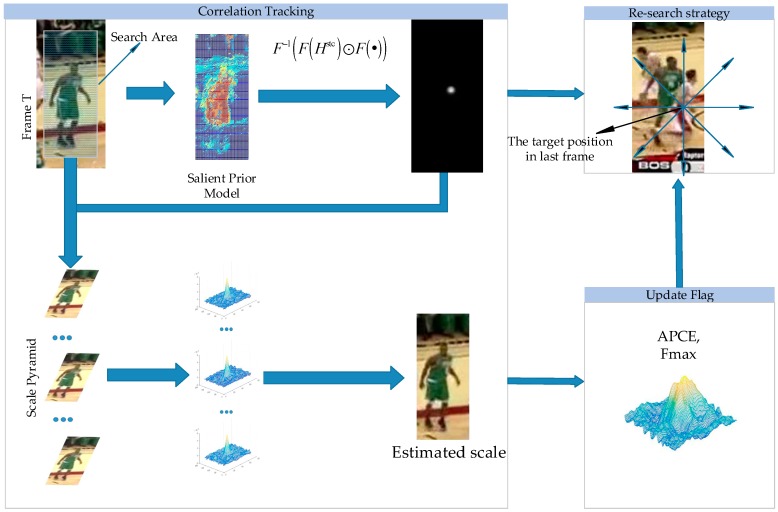
The flowchart of the proposed tracker. The salient prior model based on color distribution is used for tracking in our tracker. We also employ a scale pyramid for scale estimation. The update flag determines whether to update the model and implement the re-search strategy.

The STC tracker has a similar working framework as the other CF-based trackers which take advantage of Fast Fourier operations to achieve high running speeds, while unlike the other algorithms, the STC tracker formulates the spatial-temporal relationships between the object and its locally dense contexts into the Bayesian framework to model the statistical correlation between the features from the targets and their surrounding regions. Nevertheless there still exist some shortcomings in the standard STC tracker. Firstly, in order to guarantee the running speed, the STC tracker chooses low-level grayscale features to represent the target, which makes STC tracker not perform well when occlusion or deformation happens. Herein we point out the inherent drawbacks of the original scale adaption scheme in STC tracker and propose a simple but efficient scale pyramid to handle the scale variations. Besides that, we explore a high confidence model updating mechanism and re-search method by introducing a novel tracking indicator to avoid model corruption and predict tracking failures in advance. The process of our algorithm is shown in Figure 2 and the main procedures will be explained in detail below.

## 3. Proposed Method

In this section, we first review the STC tracker process. Then we will present our tracker (CDT) focusing on four aspects, which are the color distribution-based prior model, scale adaption scheme, high confidence updating strategy and the re-search technique.

### 3.1. Spatio-Temporal Context Tracking

In this subsection, we will introduce the principle of the STC tracker. The STC tracker formulates the tracking problem by finding the maximum of the response map which is calculated by the convolution of the prior possibility with the image. STC tracker does optimizing instead of training samples. It first learns a likelihood distribution which is the prior possibility of object location. The confidence map estimating the object location likelihood is calculated by:

(1) $$c\left(x\right)=P\left(x|o\right)=be^{-{\left|\frac{x-x^{*}}{\alpha }\right|}^{\beta }}$$

where $x\in R^{2}$ is an object location and o denotes object present in the frame. b is a normalization constant, α is a scale parameter and β is a shape parameter. $X^{c}=\left\{c\left(z\right)=\left(I\left(z\right),z\right)|z\in \Omega_{c}\left(x^{*}\right)\right\}$ is defined as a context feature set. I(z) is the image intensity at location z and $\Omega_{c}\left(x^{*}\right)$ denotes the neighborhood of location $x^{*}$. In the confidence map c(x), the location of the target is the position where P(x|o) is max. The likelihood function can be computed by:

(2) $$c\left(x\right)=P\left(x|o\right)=\sum_{c\left(z\right)\in X^{c}}P\left(x,c\left(z\right)|o\right)=\sum_{c\left(z\right)\in X^{c}}P\left(x|c\left(z\right),o\right)P\left(c\left(z\right)|o\right)$$

In this equation, P(x|c(z),o) represents the spatial relationship between the target and its context information. P(c(z)|o) is a context prior probability and is used to describe the appearance of the local context.

The conditional probability function P(x|c(z),o) can be computed by:

(3) $$P\left(x|c\left(z\right),o\right)=h^{sc}\left(x-z\right)$$

In Equation (3), $h^{sc}\left(x-z\right)$ is a function about relative distance and direction of the target position *x* and the local context position *z*. It encodes the spatial relationship between the target and its spatial context. And it will update in the process of tracking.

P(c(z)|o) can be written in the following form:

(4) $$P\left(c\left(z\right)|o\right)=I\left(z\right)\omega_{\sigma }\left(z-x^{*}\right),$$

where I(z) is image intensity that describes the appearance of the context in location *z*. $\omega_{\sigma }\left(\cdot \right)$ is a weighted function. When the location *z* is near the location *x*, the value of $\omega_{\sigma }\left(\cdot \right)$ is big and its value is obtained by the following equation:

(5) $$\omega_{\sigma }\left(z\right)=ae^{-\frac{{\left|z\right|}^{2}}{\sigma^{2}}}$$

In Equation (5), σ is a scale parameter and a is a normalization constant that restricts P(c(z)|o) to a range from 0 to 1.

Equation (2) can be written as the follows, according to Equations (3)–(5):

(6) $$\begin{array}{l} c\left(x\right)=be^{-{\left|\frac{x-x^{*}}{\alpha }\right|}^{\beta }}\\ =\sum_{Z\in \Omega_{C}(x^{*})}h^{sc}\left(x-z\right)I\left(z\right)\omega_{\sigma }\left(z-x^{*}\right)\\ =h^{sc}\left(x\right)⊗\left(I\left(x\right)\omega_{\sigma }\left(x-x^{*}\right)\right) \end{array}$$

⊗ denotes the convolution operator. To reduce the computational cost, the STC tracker does the calculation in the Fourier domain. Then we can get Equation (7):

(7) $$F\left(be^{-{\left|\frac{x-x^{*}}{\alpha }\right|}^{\beta }}\right)=F\left(h^{sc}\left(x\right)\right)⊙F\left(I\left(x\right)\omega_{\sigma }\left(x-x^{*}\right)\right),$$

where F denotes the FFT function and ⊙ is the element-wise product. Hence, we can get the value of the model $h^{sc}$ by the equation:

(8) $$h^{sc}\left(x\right)=F^{-1}\left(\frac{F\left(be^{-{\left|\frac{x-x^{*}}{\alpha }\right|}^{\beta }}\right)}{F\left(I\left(x\right)\omega_{\sigma }\left(x-x^{*}\right)\right)}\right)$$

In the *t*+1-th frame, the spatio-temporal context model is updated by:

(9) $$H_{t+1}^{stc}=\left(1-\rho \right)H_{t}^{stc}+\rho h_{t}^{sc}.$$

After that, the confidence map is calculated by:

(10) $$c_{t+1}\left(x\right)=F^{-1}\left(F\left(H_{t+1}^{stc}\left(x\right)\right)⊙F\left(I_{t+1}\left(x\right)\omega_{\sigma_{t}}\left(x-x^{*}\right)\right)\right).$$

The location of the target in the *t*+1-th frame is the place where the maximum value is in the confidence map.

### 3.2. Prior Model with Color Distribution

Grayscale features which are the simplest features in tracking methods regard the pixel values as features. For this reason, they cannot fully display the prior model and may be not robust when illumination changes. Wang et al. [2] verified that the feature extractor is one of the most important parts of any tracker. A robust feature can greatly improve the performance of a tracker. Color features are a befitting feature that can provide enough information for our tracker without much additional computational cost. Xu et al. [20] improved STC by applying a linguistic language which is an 11 dimensional color representation, instead of grayscale features. However, this approach is quite computationally expensive. Therefore, our algorithm makes use of a color histogram as feature. Given a rectangular object region O (initial bounding box or current tracker candidate) and its surrounding region S, we can apply Bayes’ rule to obtain the object likelihood at location *x* as:

(11) $$P\left(x\in O|O,S,b_{x}\right)\approx \frac{P\left(b_{x}|x\in O\right)P\left(x\in O\right)}{P\left(b_{x}|x\in S\right)P\left(x\in S\right)}$$

$b_{x}$ denotes the bin assigned to the color components of I(x). Each bin is composed of 16 pixels which are in a 4 × 4 grid. I is the input image.

From Equation (4) we can find that STC tracker only uses image intensity to represent the appearance of the context, so we replace the intensity feature by a color distribution. Let $H_{\Omega }^{I}\left(b_{x}\right)$ denote the *b*-th bin of the non-normalized histogram H computed over the region Ω∈I. Besides, the Equation (6) can be estimated directly by color histograms, i.e., $P\left(b_{x}|x\in O\right)\approx \frac{H_{O}^{I}\left(b_{x}\right)}{\left|O\right|}$ and $P\left(b_{x}|x\in S\right)\approx \frac{H_{S}^{I}\left(b_{x}\right)}{\left|S\right|}$. |⋅| means the cardinality. We can also estimate the prior probability as $P\left(x\in O\right)\approx \frac{\left|O\right|}{\left|O\right|+\left|S\right|}$. Then, the calculation formula can be simplified to:

(12) $$P\left(x\in O|O,S,b_{x}\right)=\{\begin{array}{cc} \frac{H_{O}^{I}\left(b_{x}\right)}{H_{S}^{I}\left(b_{x}\right)}, & \mathrm{if}\;I\left(x\right)\in I\left(O\cup S\right)\\ 0.5, & \mathrm{otherwise} \end{array}$$

For those unseen pixel values, both the numerator and the denominator in Equation (12) are zero, which could not provide the specific probability. Hence, we have to assign the prior probability for the unseen pixel-values according to the uniform distribution assumption, by setting it to 0.5. This discriminative model can help us to distinguish object and background pixels. This discriminative model is shown in Figure 1c.

In the baseline tracker, the context prior model P(c(z)|o) uses I(z) for calculation . While in our algorithm, with I(z) replaced by $P\left(x\in O|O,S,b_{x}\right)$, Equation (4) would be written as:

(13) $$P\left(c\left(x\right)|o\right)=P\left(x\in O|O,S,b_{x}\right)\omega_{\sigma }\left(x-x^{*}\right)$$

Furthermore, the STC tracker calculates c(x) in the frequency domain so that the Fast Fourier Transform (FFT) algorithm can be used for fast convolution. We adopt the same procedure as the previous work, and the equation is transformed as:

(14) $$F\left(c\left(x\right)\right)=F\left(h^{sc}\left(x\right)\right)⊙F\left(P\left(x\in O|O,S,b_{x}\right)\omega_{\sigma }\left(x-x^{*}\right)\right)$$

where F denotes the FFT function and ⊙ is the element-wise product. Therefore, we can obtain an equation:

(15) $$h^{sc}\left(x\right)=F^{-1}\left(\frac{F\left(c\left(x\right)\right)}{F\left(P\left(x\in O|O,S,b_{x}\right)\omega_{\sigma }\left(x-x^{*}\right)\right)}\right),$$

where $F^{-1}$ denotes the inverse FFT function.

In the *t*+1-th frame, the target location is obtained by maximizing the new confidence map:

(16) $$x_{t+1}^{*}=\underset{x\in \Omega_{c}\left(x_{t}^{*}\right)}{\arg\max}c_{t+1}\left(x\right),$$

where $c_{t+1}\left(x\right)$ is calculated by:

(17) $$c_{t+1}\left(x\right)=F^{-1}\left(F\left(H_{t+1}^{stc}\left(x\right)\right)⊙F\left(P_{t+1}\left(x\in O|O,S,b_{x}\right)\omega_{\sigma_{t}}\left(x-{x_{t}}^{*}\right)\right)\right).$$

With the number of frames changed, the spatio-temporal context is updated by:

(18) $$H_{t+1}^{stc}=(1-\rho )H_{t}^{stc}+\rho h_{t}^{sc}$$

where ρ is a learning parameter and $h_{t}^{sc}$ is obtained by Equation (15) at the *t*-th frame. We set the learning parameter as 0.075 which is the same with our baseline tracker (STC).

It should be mentioned that, we advocate a high confidence updating strategy for the baseline STC tracker, which updates the template as Equation (18) when the tracking result is accurate. We discuss this in details in Section 3.4.

### 3.3. Scale Adaptation Mechanism

In practical tracking applications, the target often undergoes scale variation. A good scale adaption mechanism could enhance the tracking performance significantly. In our baseline tracker, STC uses a scale parameter σ to describe the changes of the target scale. To be more specific, the scale parameter is updated by:

(19) $$\{\begin{array}{c} s_{t}^{\prime }=\sqrt{\frac{c_{t}\left(x_{t}^{*}\right)}{c_{t-1}\left(x_{t-1}^{*}\right)}},\\ {\bar{s}}_{t}=\frac{1}{n}\sum_{i=1}^{n}s_{t-i}^{\prime },\\ s_{t+1}=\left(1-\lambda \right)s_{t}+\lambda \bar{s_{t}},\\ \sigma_{t+1}=s_{t}\sigma_{t}, \end{array}$$

where $c_{t}\left(\cdot \right)$ is the confidence map that is computed by Equation (12), and $s_{t}^{\prime }$ is the estimated scale between two consecutive frames. $\bar{s_{t}}$ denotes the average of the estimated scales from n consecutive frames and λ>0 is a fixed filter parameter.

We can find that the STC tracker regards the ratio of the maximum response in two consecutive frames as the scale parameter. In CF-based trackers, the response is obtained by calculating the cross correlation between the input target samples and the model template. However, since the target often undergoes complicated tracking conditions like illumination variation, deformation and occlusion, the response value may change abruptly even when the target has no scale changes. Therefore, we abandon the scale estimation in traditional the STC tracker which is inconsequential. Our algorithm applies a simple but effective method called scale pyramid to estimate the scale variation.

We enlarge the target by a different multiple to find the best scale conversion factor. We set a series of fixed multiple. The template size is $s_{T}=\left(s_{x},s_{y}\right)$ and the scaling pool is defined as $S=\left\{t_{1},t_{2},\ldots ,t_{k}\right\}$. We suppose that the target window size is $s_{t}$ in the original image space. For the current frame, we sample k sizes in $\left\{t_{i}s_{t}|t_{i}\in S\right\}$ to find the proper target. Note that the convolution operation needs the template data with fixed size. After sampling k sizes, we resize the samples into the fixed size $s_{T}$. Finally, the response map is calculated by:

(20) $$\arg\max c_{t+1}\left(x,t_{i}\right)$$

### 3.4. Tracking Update Strategy

In the STC tracker, the template model is updated in every frame regardless of whether the tracking result is accurate or not. Some other trackers use a strategy of updating every several frames. Doing so can reduce the cumulative error to a certain extent. All the above measures may cause model corruption problems when the target is detected inaccurately, severely occluded or totally missing. In our algorithm, we adopt a new update strategy using the feedback from the results to decide whether to update the model and the parameters.

As discussed above, we find that the response map, not only the peak value but also the shape, would fluctuate fiercely when the target undergoes severe occlusion or deformation, etc. In other words, the peak value and the fluctuation can reveal the accuracy of the tracking result. Considering this, our method takes into account both the peak value and the fluctuation of the response map as indicators. The peak value $F_{\max}$ denotes the maximum response score of the response map. To measure the fluctuation of the response map we adopt the same criterion as Wang et al. proposed in [21] called average peak-to-correlation energy (APCE), which is defined as:

(21) $$APCE=\frac{{\left|F_{\max}-F_{\min}\right|}^{2}}{mean\left(\sum_{\omega ,h}{\left(F_{\omega ,h}-F_{\min}\right)}^{2}\right)}$$

where $F_{\max}$, $F_{\min}$ and $F_{\omega ,h}$ denote the maximum, minimum and the ω-th row h-th column elements of the detection response map.

If the tracking result in the current frame is high-confidence, $F_{\max}$ and APCE of the current frame should be greater than their respective historical average values with ratios $\beta_{1}$ and $\beta_{2}$. Only when the results satisfy both the APCE and $F_{\max}$ standards at the same time, will our update strategy be implemented. We test our algorithm in some challenging sequences. The result shows our update strategy is effective and can maximize the introduction of errors in the model when the occlusion occurs.

### 3.5. Re-Search Target Measure

Most of the existing correlation tracking methods focus on short-term tracking without a re-detection mechanism. If the object is completely occluded, the tracker without re-detection could hardly recover from the model drift and track accurately any more. As we discussed in Section 3.4, the APCE and $F_{\max}$ criteria can reveal the accuracy of the tracking. Benefitting from this, we can use these two criteria as indicators to infer whether there is occlusion. When the target is occluded in our algorithm, the tracker won’t update the model according to our update strategy. At the same time, our tracker will re-search for the target around the target location of the previous frame.

When occlusion occurred, the target may move in any direction. Hence, we need to re-search for the target in the current frame, otherwise the tracker will be out of action. In order to reduce the computational cost, we divide the 360 degrees into eight sectors to re-search the object. We then search a coarse region from these eight directions around the center of the latest location by calculating the cross-correlation of the template and each sample. Considering that the movement of the target in each frame is continuous, we move the searching area of the previous frame in eight directions to get the coarse region. The coarse region of each direction is the same size with our original searching region. We would compare the max peak response value in each direction. The direction of the maximum value indicates the target movement after occlusion. This re-search measure was tested in our data set and the experimental results prove that it is useful for resolving the problem of occlusion. The brief process of the proposed CDT is shown in Algorithm 1.
**Algorithm 1** Our proposed tracking method1: Initial the target box in the first frame
$b=\left[x_{0},y_{0},h_{0},w_{0}\right]$ and other parameters.2: Initial the salient prior, the context prior model and the spatial context model.3: **For**
frame=2,3,… until the last frame.4: Crop out the context window and the target window, then compute the object likelihood $P\left(x\in O|O,S,b_{x}\right)$ via the salient prior by Equation (12).5: **If** frame > 1,6: Calculate the confidence map $c_{t}\left(x,t_{i}\right)$ with scale pyramid to find the proper scale variation $c_{t}\left(x\right)$ according to Equation (17).7: Compute the update criteria to update the update flag.8: **If** the update flag is not 1,9: Re-search the target around the location where the target was in the last frame.10: Find the max response map in different directions.11: **End**12: **End**13: Locate the target by maximizing the response map in the current frame.14: **If** the update flag = 1,15: Update the salient prior.16: Compute the context prior model and the spatial context model.17: Update the spatio-temporal model by Equation (18).18: **End**.19: **End**.

## 4. Experiments

We evaluated the performance of our proposed method CDT on 29 challenging sequences provided on OTB2015, which involves most of the affecting factors during tracking procedure. This algorithm is compiled on MATLAB R2015b using the MATLAB language. The configuration of our computer is an Intel i5-4590 CPU (3.30 GHz), 8 GB RAM memory and the Win 7 operating system. We tested the proposed method against 12 state-of-the-art trackers, including TLD [11], KCF [15], Struck [13], L1APG [22], ASLA [23], IVT [5], KMS [24], CSK [19], OAB [25], MIL [9], CT [12], STC [1]. The parameters in our algorithm are fixed for all the experiments. For the other state-of-the-art trackers, we employ their original provided code or binary code in which the parameters of each tracker are adjusted to the best value for the sequences. Our method runs at 60 frames per second on the MATLAB platform. After a lot of practice, we set $\beta_{1}$ and $\beta_{2}$ as 0.3 and 0.5. In order to balance between speed and effect of scale pyramid, we set the value of k as 7 after a lot of tests. And we set S={1,0.985,0.99,0.995,1.005,1.01,1.015} in our experiments. When re-searching the target, the coarse region is obtained by moving the original searching area of the previous frame by one fifth of the height or the width of the original area. The moving distance is set as one fifth of the width of the original searching area on the *X* axis. On the *Y* axis, the moving distance is set as one fifth of the height of the original searching area.

### 4.1. Qualitative Evaluation

To better analyze the effectiveness and robustness of the proposed tracker, in this subsection we mainly discuss the performance over most common challenging factors, namely occlusion, deformation, scale variation and rotation.

#### 4.1.1. Occlusion

**Figure 3:**
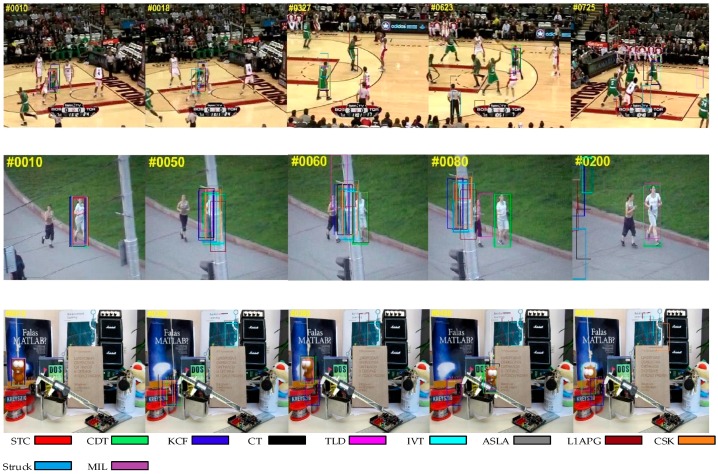
Qualitative results of our method and the nine state-of-the-art tracking methods on sequences *Basketball*, *Jogging-2* and *Lemming*. In these sequences, the targets undergo occlusion.

Figure 3 shows the situation in which the target is partially or short-term fully occluded. We can find that some trackers such as IVT somehow fail in the sequences, while our algorithm tracks the target effectively in these sequences. The reason why our algorithm can outperform than other trackers is that we exploit a new update strategy and a re-search mechanism. When the problem of occlusion occurs, the criteria in our method will reveal it and the model won’t be updated to avoid introducing errors. In addition, our method can re-search the target after losing the target when occlusion occurs. These make our algorithm more robust and effective.

#### 4.1.2. Background Clutter

**Figure 4:**
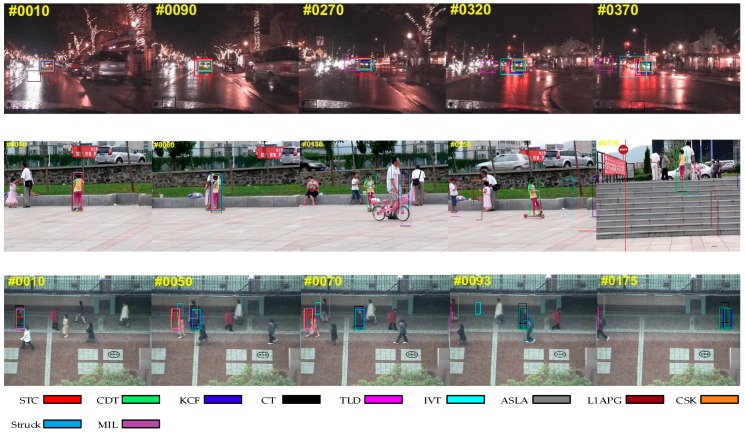
Qualitative results of our method and the nine state-of-the-art tracking methods on sequences *Cardark*, *Girl2* and *Subway*. In these sequences, there is a background clutter problem.

In Figure 4, the targets are difficult to distinguish from the background. With background clutter, the target window may drift onto the background. While we can see that our algorithm is better than other trackers in handling this problem. This could be attributed to the usage of a color distribution prior model, which enhances the distinguishing ability for appearance modeling.

#### 4.1.3. Scale Variation

**Figure 5:**
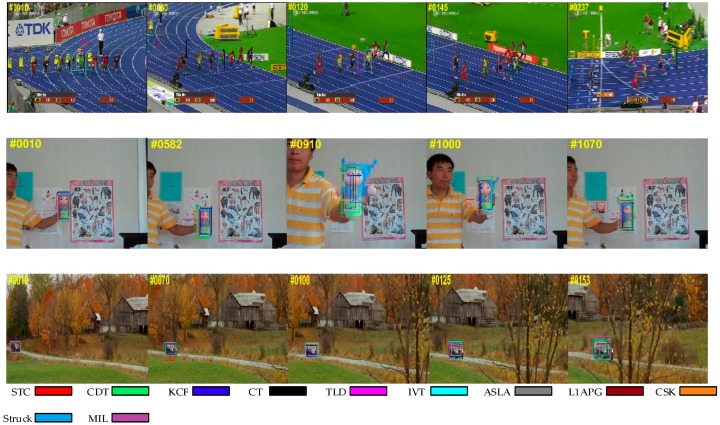
Qualitative results of our method and the nine state-of-the-art tracking methods on sequences *Bolt*, *Carscale* and *Doll*. In these sequences, the targets undergo scale variation.

Figure 5 presents the tracking results on the sequences with scale variation. The scale of the target usually changes during tracking. Therefore, it is necessary for a tracker to adjust the size of the target window, otherwise, the tracker may fail because it acquires more background information. As we utilize a scale pyramid for scale adaptation, our tracker can capture the target with different scales for further selection. Some other trackers, such as CT and TLD, cannot adapt to scale variation.

#### 4.1.4. Rotation

**Figure 6:**
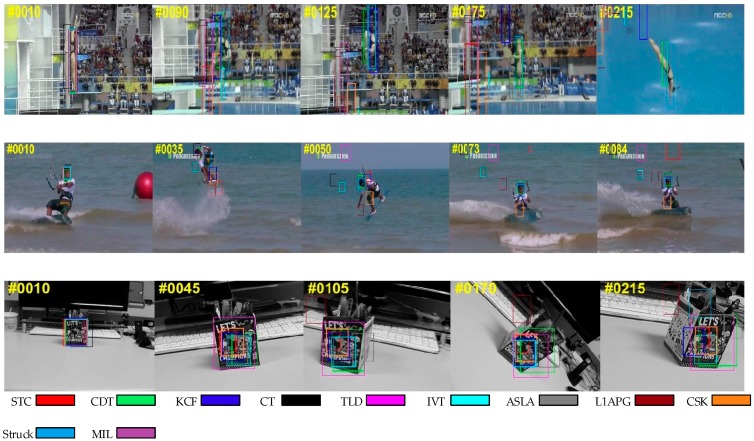
Qualitative results of our method and the nine9 state-of-the-art tracking methods on sequences *Diving*, *Vase* and *Kitesurf*. In these sequences, the targets undergo rotation.

In Figure 6, the problem of rotation affects the appearance of the target. Rotation usually occurs when the target moves or the viewpoint changes. Many trackers drift when this occurs while our algorithm tracks the target to the end. This benefits from the use of color distribution as a feature which is robust when there is rotation.

### 4.2. Quantitative Evaluation

In this subsection, we apply One-Pass-Evaluation, which is a common evaluation method used in OTB proposed in [26] to evaluate the performance of the proposed tracker and other related trackers. Two criteria, success rate and precision, are used for quantitative evaluation, which are defined as follows.

#### 4.2.1. Precision

The percentage of the frames whose center location error are less than the predefined threshold. While the center location error indicates the distance between the center of the tracking results and the one of the bounding box. In this experiment, 20 pixels are used to rank these trackers.

#### 4.2.2. Success Rate

The percentage of frames where the overlap rates of the tracking region and bounding box are larger than the threshold. The area under the curve (AUC) indicates the tracking performance throughout all the thresholds vary from 0 to 1.

#### 4.2.3. Overall Performance

**Figure 7:**
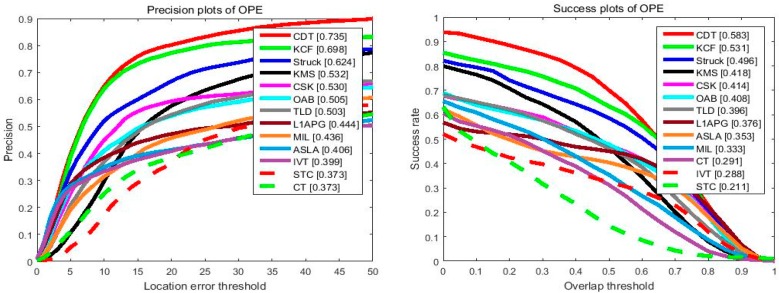
The precision plots and success plots of OPE for 13 trackers. The performance score of each tracker is shown in the figure. Best viewed on color display.

Figure 7 illustrates the overall performance of the 13 trackers in terms of the mentioned criteria. One can see that the proposed method ranks first on success plot and precision plot. In the success plot, the proposed algorithm achieves the AUC of 0.583, which outperforms the baseline STC tracker by 37%, which could be attributed to its more powerful and distinguished features. Meanwhile, in the precision plot, our tracker also achieves better performance than the KCF tracker and Struck tracker, which yield the best performance in VOT2014 and OTB2013. We should point out both the mentioned trackers focus on short-term tracking, which could hardly recover from corruption problems caused by updating and other challenging factors, while, with the help of a high-confidence updating scheme and re-detection strategy, the proposed tracker has significantly enhanced tracking performance.

#### 4.2.4. Attribute-Based Performance

**Figure 8:**
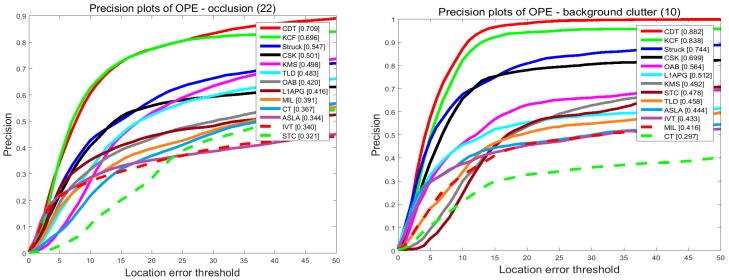

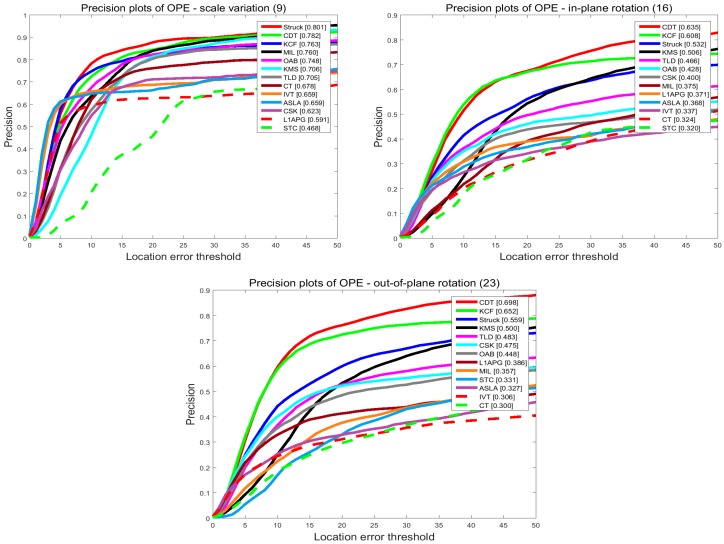
The precision plots of sequences with occlusion, background clutter, scale variation and rotation. The performance score of each tracker is shown in the figure. Best viewed in color display.

**Figure 9:**
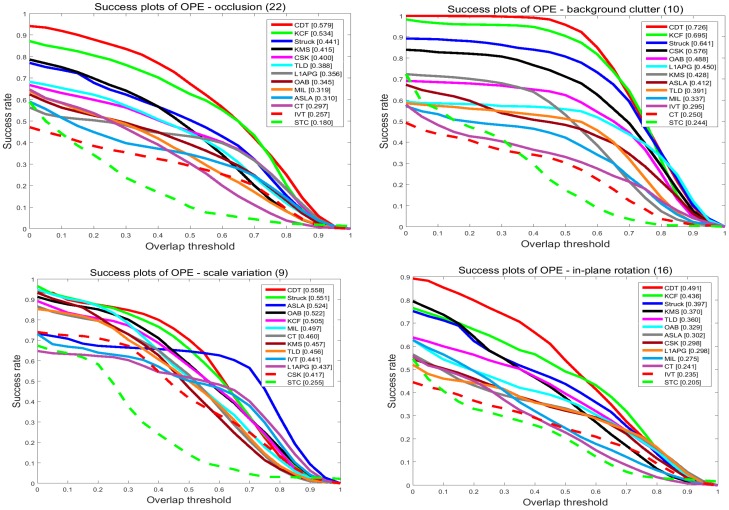

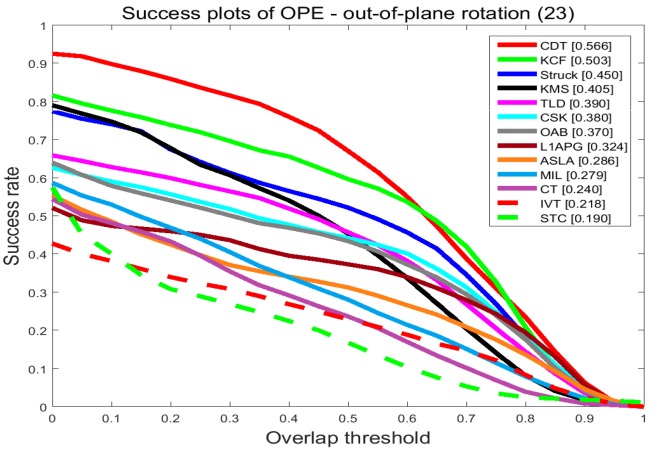
The success plots of sequences with occlusion, background clutter, scale variation and rotation. The performance score of each tracker is shown in the figure. Best viewed in color display.

In order to investigate the effectiveness of the proposed tracker, we compare our tracker and the others in terms of the attributes listed in the last subsection. The results are shown in Figure 8 and Figure 9. On the videos with occlusion, our tracker ranks first among the evaluated trackers. In precision plots, CDT achieves 0.709, outperforming KCF by 1.3%. In success plots, CDT is 0.579, which is higher than KCF. Occlusion may pollute the target model and make it hard to extract features. KCF uses the HOG feature to describe the target which is stable when occlusion occurs. Though the proposed CDT exploits color distribution as a feature, we have a re-search method and an update strategy to overcome occlusion.

On the videos with background clutters and rotation, our tracker ranks first among these trackers. In the precision plots of background clutters, our tracker CDT achieves 0.882 that is far better than KCF. For in-plane rotation and out-plane rotation, CDT scores 0.635 and 0.698 which outperforms KCF which scored 0.608 and 0.652, by 3%. In the success plots of background clutters, CDT ranks first with a score of 0.726. CDT, that scores 0.491 and 0.566 in in-plane rotation and out-plane rotation outperforms KCF by 6%. The proposed CDT utilizes color distribution that is not much changed in the rotation to describe the target. Besides that, our tracker has a salient prior model to reduce the influence of background.

On the videos with the problem of scale variation, CDT ranks second among these evaluated trackers with a narrow margin to the best tracker Struck. Struck ranks first in these trackers with 0.8 scores in the precision plots, while the proposed CDT scores 0.782 which is only 2% less. In the success plots of scale variation, CDT outperforms Struck by 0.7%. Struck makes use of structured output SVM which extends the output space to include scale variation. As CDT adopts a scale pyramid, it also achieves a good performance.

### 4.3. Demonstrations

To evaluate the effect of update strategy and re-search measure, additional comparison experiments are conducted on the OTB 2015 benchmark. CDT is our proposed method. Only F-max is the method which is the same as CDT except it only uses F-max as update criterion and Only APCE indicates the method that is the same as CDT except for only using APCE as update criterion. No update indicates the method without update strategy. No re-search means it doesn’t have a research measure. All of these methods mentioned above are based on CDT and utilize the same parameters and test sequences.

#### 4.3.1. Demonstrations of Update Strategy

**Figure 10:**
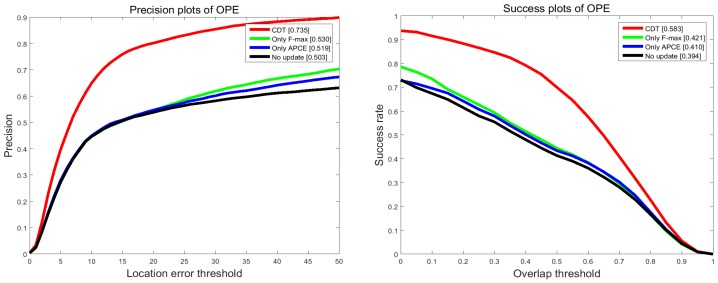
Tracking performance of CDT, Only F-max, Only APCE and No update on OTB 2013 dataset.

To analyze to what extent the update strategy improves the tracking performance, we conduct comparison experiments between CDT, No update, Only F-max and Only APCE, as shown in Figure 10. It is obvious that CDT leads to 23.2% and 19.9% performance improvements in terms of precision and success rate. Besides, it can be seen that the improvement of only using one criterion is limited. Using two criteria enhances the performance dramatically.

#### 4.3.2. Demonstrations of Re-Search Measure

**Figure 11:**
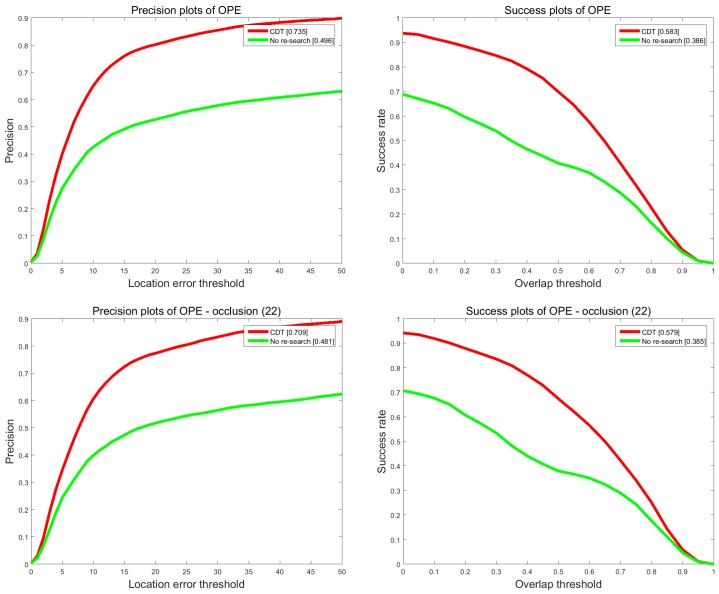
Tracking performance of CDT and No re-search on OTB 2013 dataset.

To investigate the effect of the re-search measure to the tracking performance, we do another comparison experiment between CDT and No re-search. The results are shown in Figure 11. We can see that re-search measure can significantly enhance the performance of handling occlusion. For the precision and success rate, re-search measure can lead to 23.9% and 19.7% performance improvements.
